# Pirfenidone Sensitizes Hepatic Stellate Cells to Ferroptosis by Reprogramming Glutamine and Serine Metabolism for GSH Depletion

**DOI:** 10.3390/antiox15050552

**Published:** 2026-04-26

**Authors:** Jia Li, Li Wang, Yakun Li, Junyu Wang, Manon Buist-Homan, Klaas Nico Faber, Han Moshage

**Affiliations:** 1 Department of Gastroenterology and Hepatology, University Medical Center Groningen, University of Groningen, 9713 GZ Groningen, The Netherlands; 2 Department of Pathology and Medical Biology, University Medical Center Groningen, University of Groningen, 9713 GZ Groningen, The Netherlands

**Keywords:** pirfenidone, hepatic stellate cell, liver fibrosis, metabolic reprogramming, ferroptosis

## Abstract

Pirfenidone (PFD) shows therapeutic potential for liver fibrosis, but its molecular mechanisms are not fully elucidated. Activation of hepatic stellate cells (HSCs) is central to liver fibrosis, making their targeted elimination a prime therapeutic strategy. Since amino acid metabolism governs both HSC activation and ferroptosis, we investigated whether PFD acts by reprogramming these metabolic pathways. Analysis of primary rat HSCs revealed that their in vitro activation induced fibrotic markers, including collagen type I and α-smooth muscle actin, as well as key metabolic enzymes. Specifically, we observed upregulation of glutaminase 1, initiating glutaminolysis to produce glutamate; serine hydroxymethyltransferase 2, which generates glycine from serine; and pyrroline-5-carboxylate synthase, the rate-limiting enzyme for de novo proline synthesis. Treatment with PFD suppressed HSC activation by reducing protein levels of these enzymes, an effect consistent with PFD’s inhibition of activating transcription factor 4 nuclear accumulation. This created a dual metabolic vulnerability, limiting amino acid precursors for both collagen synthesis and the master antioxidant glutathione (GSH). Consequently, while PFD alone was not cytotoxic, GSH depletion sensitized activated HSCs to ferroptosis. Co-treatment with the ferroptosis inducer erastin triggered a synergistic increase in reactive oxygen species, labile iron, and lipid peroxidation, culminating in cell death. This synergistic lethality was abrogated by the ferroptosis inhibitor ferrostatin-1 and the antioxidant N-acetylcysteine, confirming ferroptosis as the specific cell death modality. Our study uncovers a dual anti-fibrotic mechanism for PFD: PFD inhibits collagen synthesis by limiting key amino acid precursors and depletes GSH. This compromises antioxidant defenses, creating vulnerability to ferroptosis. Our findings establish a rationale for using PFD in combination therapies designed to eliminate activated HSCs.

## 1. Introduction

Liver fibrosis, the common pathological endpoint of most chronic liver diseases, is a primary driver of global morbidity and mortality. End-stage liver disease and its associated complications are collectively responsible for approximately two million deaths annually worldwide. The hallmark of fibrosis is the progressive accumulation of extracellular matrix (ECM) components, a process orchestrated by a heterogeneous population of fibrogenic cells. This fibrogenic population is composed mainly of activated hepatic stellate cells (aHSCs), which undergo profound activation, proliferation, and transdifferentiation [1]. Due to their central role, strategies aimed at eliminating aHSCs or promoting their inactivation are considered promising therapeutic approaches [2,3].

The proliferative and migratory capacities of ECM-secreting aHSCs demand substantial energy input, triggering profound metabolic reprogramming [4]. Mounting evidence indicates that these metabolic shifts are not merely consequences of HSC activation but are intrinsically required for their activation and differentiation [5,6]. Notably, aHSCs exhibit a pronounced dependency on glutamine. This reliance is manifested through heightened glutaminolysis, where glutamine is converted into glutamate by glutaminase 1 (GLS1) to fuel the tricarboxylic acid cycle. This metabolic signature is consistently observed in cultured HSCs, experimental models of liver injury, and liver samples from patients with advanced fibrosis [7,8,9]. Furthermore, glutamine serves as a crucial precursor for proline synthesis via pyrroline-5-carboxylate synthase (P5CS), directly supplying the building blocks for collagen [10,11]. Similarly, the de novo synthesis of glycine, the most abundant amino acid in collagen, is catalyzed by serine hydroxymethyltransferase 2 (SHMT2) from serine [12]. This enzyme is found to be significantly upregulated in HSCs of fibrotic mouse livers [13]. Collectively, these findings establish metabolic reprogramming, particularly of amino acid pathways, as a critical axis for sustaining HSC activation.

Beyond fueling bioenergetics and biosynthesis, emerging studies reveal that amino acid availability is also intricately linked to ferroptosis. This regulated form of cell death is a key determinant of HSC survival and a potential mechanism for their elimination [14,15,16]. Ferroptosis is an iron-dependent process driven by the accumulation of lipid peroxides, often precipitated by glutathione (GSH) depletion [17]. The roles of GLS1-mediated glutaminolysis and SHMT2-driven serine metabolism extend to regulating the cellular redox state, as their respective products, glutamate and glycine, are indispensable precursors for the synthesis of the master antioxidant GSH [18,19]. Indeed, inducing ferroptosis in HSCs has been shown to ameliorate fibrosis, highlighting this pathway as a promising therapeutic target [20,21,22].

Pirfenidone (PFD) is an orally available small-molecule drug clinically approved for the treatment of idiopathic pulmonary fibrosis (IPF). While it demonstrates broad antifibrotic and anti-inflammatory activities, its precise mechanisms of action are not fully understood [23,24,25]. Its clinical efficacy has spurred investigations into its potential for other fibrotic conditions, and several trials are currently evaluating its use in patients with liver fibrosis [26,27].

Despite its emerging clinical relevance for liver disease, the direct effects of PFD on the metabolic vulnerabilities of HSCs remain unexplored. This study was designed to bridge that knowledge gap. We hypothesized that PFD exerts its antifibrotic effects by directly remodeling the amino acid metabolic network in HSCs, thereby suppressing their activation, curtailing collagen synthesis, and concurrently sensitizing them to ferroptosis through GSH depletion. Utilizing primary rat HSCs undergoing spontaneous activation in vitro, we investigated the impact of PFD on glutamine and serine metabolism and the cellular susceptibility to ferroptosis. Our objective was to elucidate the precise molecular mechanisms linking the therapeutic action of PFD to the metabolic regulation of HSC fate.

## 2. Materials and Methods

### 2.1. Reagents and Chemicals

Pirfenidone (PFD), erastin (ERA), and ferrostatin-1 (Fer-1) were purchased from MedChemExpress (Sollentuna, Sweden). Tunicamycin, 4-phenylbutyrate (4-PBA), and N-acetyl-L-cysteine (NAC) were obtained from Sigma-Aldrich (Zwijndrecht, The Netherlands). The GLS1 inhibitor CB-839 was purchased from Selleckchem (Houston, TX, USA).

### 2.2. Primary Rat Hepatic Stellate Cell Isolation, Culture, and Treatment

Male Wistar rats (250–300 g) were procured from Charles River Laboratories (Wilmington, MA, USA) and housed at the University Medical Center Groningen Central Animal Facility for a one-week acclimatization period. Animals were maintained under controlled conditions (25 ± 2 °C, 12 h light/dark cycle) with ad libitum access to food and water. To ensure full surgical anesthesia and minimize suffering, rats were first induced with 5% isoflurane inhalation for 5 min, followed by an intraperitoneal injection of ketamine (100 mg/mL, 60 mg/kg) and medetomidine hydrochloride (1 mg/mL, 0.5 mg/kg). Primary rat HSCs were isolated using a two-step collagenase perfusion method as previously described [28]. Briefly, the liver was perfused with Pronase E (Merck, Amsterdam, The Netherlands) followed by collagenase P (Roche, Almere, The Netherlands). The resulting cell suspension was layered onto a 13% Nycodenz (Axis-Shield, Oslo, Norway) density gradient, and HSCs were purified by density gradient centrifugation. Freshly isolated HSCs were seeded onto culture plates and maintained in a humidified incubator at 37 °C with 5% CO_2_. The complete culture medium consisted of Iscove’s Modified Dulbecco’s Medium (IMDM; Thermo Fisher Scientific, Breda, The Netherlands) supplemented with 20% fetal bovine serum, 1% MEM nonessential amino acids, 1% sodium pyruvate, and 50 μg/mL gentamycin (all from Thermo Fisher Scientific, Waltham, MA, USA), as well as 100 U/mL penicillin, 100 U/mL streptomycin, and 250 ng/mL Fungizone (all from Lonza, Basel, Switzerland). For treatment experiments, HSCs were treated on day 1, day 3, or day 7 post-isolation, corresponding to quiescent (qHSC), intermediately activated, and fully activated (aHSC) states, respectively. Cells were exposed to PFD (10–1000 μM) or the GLS1 inhibitor CB-839 (0.1–10 μM) for 72 h. To investigate the modulation of ferroptosis sensitivity in aHSCs, cells were treated with PFD for 72 h, with either the ferroptosis inducer ERA (10 μM), the ferroptosis inhibitor Fer-1 (1 μM), or the antioxidant NAC (2 mM) being added for the last 24 h of incubation.

### 2.3. Cell Proliferation Assays

Cell proliferation was assessed using two independent methods. First, the proliferation of aHSCs was monitored in real-time using the xCELLigence Real-Time Cell Analysis (RTCA) system (Agilent Technologies, Santa Clara, CA, USA). aHSCs were seeded into 16-well E-plates at a density of approximately 3000 cells per well. Following cell attachment, cultures were treated with or without PFD. Cell proliferation was monitored by measuring electrical impedance, which is expressed as a unitless Cell Index. To quantify the treatment effect, the Cell Index at each time point was normalized to the value at the time of drug addition, yielding the Normalized Cell Index (NCI). In parallel, cell proliferation was quantified using a bromodeoxyuridine (BrdU) cell proliferation ELISA kit (Roche, Almere, The Netherlands), according to the manufacturer’s instructions. aHSCs were seeded at an equal density into black, clear-bottom 96-well microplates. Wells containing only culture medium served as blank controls. After allowing the cells to adhere, they were subjected to the indicated drug treatments. Four hours prior to the end of the experiment, BrdU labeling solution was added to each well. Following this incubation, the labeling medium was removed, and cells were fixed and their DNA denatured by adding FixDenat solution for 30 min at room temperature. Subsequently, the FixDenat solution was discarded, and cells were incubated with an anti-BrdU-peroxidase (POD) antibody conjugate for 90 min at room temperature. The plates were then washed three times with the provided washing solution for 5 min per wash. Finally, the substrate solution was added, and after a 5 min incubation at room temperature, luminescence was measured using a BioTek Synergy H4 Plate Reader (Agilent Technologies, Winooski, VT, USA).

### 2.4. Cell Viability Assay

Cell viability was determined using the Cell Counting Kit-8 (CCK-8) assay. aHSCs were seeded into 96-well plates and subjected to the indicated treatments for 72 h. Following the treatment period, the culture medium in each well was replaced with fresh, serum-free medium. CCK-8 solution was then added, and the plates were incubated for an additional 4 h at 37 °C. The absorbance at a wavelength of 450 nm was subsequently measured using a BioTek Epoch Microplate Reader (Agilent Technologies, Winooski, VT, USA).

### 2.5. Mitochondrial Function Measurement

Mitochondrial respiration was assessed by measuring the oxygen consumption rate (OCR) using a Seahorse XF96 Extracellular Flux Analyzer (Agilent Technologies, Santa Clara, CA, USA). One day prior to the assay, aHSCs were seeded into a Seahorse XF96 cell culture microplate at a density of 15,000 cells per well. On the day of the experiment, the standard culture medium was replaced with XF DMEM medium supplemented with 10 mM glucose and 2 mM glutamine (all from Agilent Technologies, Amstelveen, The Netherlands). The plate was then incubated at 37 °C in a non-CO_2_ incubator for 1 h to allow for temperature and pH equilibration. The OCR was measured at baseline and after the sequential injection of oligomycin (2.5 μM), the uncoupler dinitrophenol (DNP; 50 μM), and a mixture of rotenone (2 μM) and antimycin A (4 μM). These measurements were used to calculate key parameters of mitochondrial bioenergetics.

### 2.6. Live-Cell Imaging of Intracellular Reactive Oxygen Species Generation and Ferrous Iron Detection

To visualize intracellular reactive oxygen species (ROS) and ferrous iron (Fe^2+^), live-cell fluorescence microscopy was performed. Following treatment, aHSCs cultured in 12-well plates were washed once with PBS. The cells were then incubated for 30 min at 37 °C in the dark with fresh, serum-free medium containing either 10 μM 2′,7′-dichlorodihydrofluorescein diacetate (H2DCFDA) for ROS detection or 1 μM FerroOrange for Fe^2+^ detection (both from MedChemExpress, Sollentuna, Sweden). Subsequently, nuclei were counterstained with Hoechst (Thermo Fisher Scientific, Bleiswijk, The Netherlands) for 10 min. Fluorescence was immediately imaged using a microscope (Leica Microsystems, Wetzlar, Germany).

### 2.7. Immunofluorescence Staining

HSCs were cultured on glass coverslips. Following the indicated treatments, cells were washed with PBS, fixed with 4% paraformaldehyde for 15 min, and then permeabilized with 0.1% Triton X-100 (Sigma-Aldrich, Zwijndrecht, The Netherlands) in PBS for 30 min at room temperature. To minimize non-specific antibody binding, cells were subsequently blocked with 1% bovine serum albumin (BSA) in PBS for 1 h. For activating transcription factor 4 (ATF4) detection, cells were pre-treated for 12 h with the ER stress inducer tunicamycin or the inhibitor 4-PBA prior to fixation and subsequent staining with an anti-ATF4 antibody (Cell Signaling Technology, Leiden, The Netherlands). After washing, cells were incubated with the appropriate Alexa Fluor-conjugated secondary antibodies (1:400). These included donkey anti-goat Alexa Fluor 594, donkey anti-mouse Alexa Fluor 488, and goat anti-rabbit Alexa Fluor 594 (all from Thermo Fisher Scientific, Bleiswijk, The Netherlands). Finally, coverslips were mounted onto slides using ProLong Gold Antifade Mountant with DAPI (Dako, Agilent Technologies, Amstelveen, The Netherlands). All images were acquired using a fluorescence microscope (Leica Microsystems, Wetzlar, Germany).

### 2.8. Reduced (GSH) and Oxidized (GSSG) Glutathione Assay

The intracellular concentrations of reduced glutathione (GSH) and oxidized glutathione (GSSG) were quantified using a commercial GSH/GSSG Assay Kit (MedChemExpress, Sollentuna, Sweden) according to the manufacturer’s protocol. Cell lysates were clarified by centrifugation, and the resulting supernatants were collected for analysis. Total glutathione (GSH + GSSG) was quantified using a kinetic enzymatic recycling assay, where the formation of a chromogenic product was monitored at 412 nm. To measure GSSG specifically, GSH was first chemically masked in a separate aliquot before the same enzymatic assay was performed. Concentrations for total glutathione and GSSG were calculated against their respective standard curves. All measurements were performed in triplicate, and the final concentrations were normalized to the total protein content of the respective sample lysates.

### 2.9. Western Blotting

Whole-cell lysates were prepared from HSCs using a lysis buffer supplemented with a cocktail of protease and phosphatase inhibitors. Protein concentrations were quantified using the DC Protein Assay Kit (Bio-Rad, Veenendaal, The Netherlands). Equal amounts of protein from each sample were separated by SDS-PAGE and then transferred onto nitrocellulose membranes (Amersham, Cytiva, Eindhoven, The Netherlands) using the Trans-Blot Turbo semi-dry transfer system (Bio-Rad). The membranes were then incubated with primary antibodies overnight at 4 °C, followed by a 1 h incubation with the appropriate horseradish peroxidase (HRP)-conjugated secondary antibodies at room temperature. Immunoreactive bands were captured with the ChemiDoc MP Imaging System (Bio-Rad). A detailed list of all antibodies used in this study is provided in Supplemental Table S1.

### 2.10. RNA Extraction and Quantitative Real-Time PCR (RT-qPCR)

Total RNA was isolated from HSCs with TRI reagent (Sigma-Aldrich, Zwijndrecht, The Netherlands). Complementary DNA was then generated from the RNA templates using M-MLV Reverse Transcriptase (Thermo Fisher Scientific). The relative abundance of target transcripts was quantified by qPCR on a QuantStudio 3 system (Thermo Fisher Scientific, Waltham, MA, USA) with specific TaqMan primers and probes as provided in Supplemental Table S2. All data were normalized to the expression of the endogenous control *36B4*, and relative expression levels were calculated via the comparative 2^−ΔΔCT^ method.

### 2.11. Bioinformatics Analysis

To elucidate the transcriptomic changes associated with HSC activation and heterogeneity, a multi-faceted bioinformatics approach was employed, utilizing three publicly available datasets from the Gene Expression Omnibus (GEO) database (https://www.ncbi.nlm.nih.gov/geo/, accessed on 16 March 2026). First, to assess the expression of specific metabolic genes, the bulk transcriptomic dataset GSE67664 was analyzed [29]. This dataset included profiles from four primary human qHSCs and aHSCs. Data was processed in R (version 4.4.3, R Foundation for Statistical Computing, Vienna, Austria) using the GEOquery package (version 2.78.0) to extract log2-transformed expression values. The relative expression levels of *GLS1*, *SHMT2*, and *ALDH18A1* were compared between the qHSC and aHSC groups using a two-tailed Student’s *t*-test. Subsequently, Gene set enrichment analysis (GSEA) was conducted using transcriptomic data from primary human qHSCs and aHSCs from the GSE68001 dataset [30]. The enrichment of the “WP_METABOLIC_PATHWAYS_OF_FIBROBLASTS” gene set was evaluated against this ranked list to calculate an Enrichment Score (ES), with statistical significance determined by the Normalized Enrichment Score (NES) and False Discovery Rate (FDR). Finally, to dissect HSC heterogeneity at single-cell resolution, we analyzed a single-cell RNA-sequencing (scRNA-seq) dataset (GSE171904) of murine HSCs from control (Oil), bile duct ligation (BDL), and carbon tetrachloride (CCl4) models using the Seurat package [31]. After identifying HSC subpopulations via unsupervised clustering (resolution = 0.3), we assessed the functional state of each subpopulation by quantifying the enrichment of the “GOBP_NEGATIVE_REGULATION_OF_FERROPTOSIS” gene set using single-sample GSEA (ssGSEA). Furthermore, differentially expressed marker genes defining each HSC subpopulation were identified using the Wilcoxon rank-sum test with stringent filtering criteria. Cluster compositions and marker expression were visualized using UMAP projections and dot plots. We further examined the expression patterns of key ferroptosis-related genes, including glutathione peroxidase 4 (*Gpx4*), ferritin heavy chain 1 (*Fth1*), Apoptosis-inducing factor mitochondria-associated 2 (*Aifm2*), and GTP cyclohydrolase 1 (*Gch1*). Violin plots were generated to visualize the distribution and heterogeneity of gene expression within each cluster, displaying median values and interquartile ranges. Integrated dot plot analyses were performed to represent mean expression levels and the fraction of expressing cells for each gene across all identified HSC subpopulations.

### 2.12. Statistical Analysis

All experiments were performed a minimum of three times using biologically independent preparations of HSCs. Quantitative data are presented as the mean ± standard deviation (SD). Statistical significance between two groups was determined using a two-tailed Student’s *t*-test. For comparisons among three or more groups, a one-way analysis of variance (ANOVA) was employed, followed by Tukey’s post hoc test for multiple comparisons. A value of *p* < 0.05 was considered statistically significant. All statistical analyses were conducted using GraphPad Prism software (Version 9.0; GraphPad Software, Inc., San Diego, CA, USA).

## 3. Results

### 3.1. Upregulation of Amino Acid Metabolism Is a Key Feature of HSC Activation

**Figure 1:**
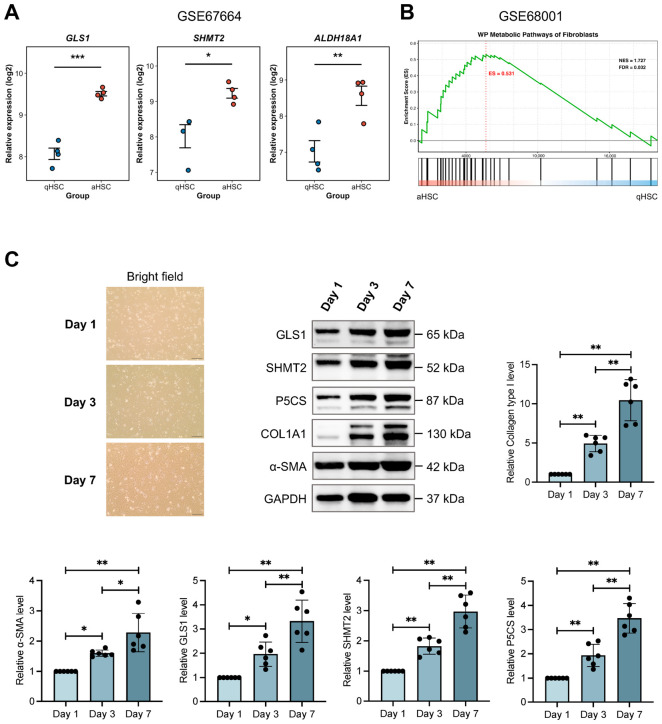
HSC activation is characterized by the upregulation of key amino acid metabolic pathways. Publicly available bulk RNA-sequencing datasets (GSE67664 and GSE68001) from primary human quiescent HSCs (qHSCs) and activated (aHSCs) were analyzed for changes in specific metabolic genes and pathways. In parallel, primary rat HSCs were cultured in vitro for 1, 3, and 7 days to represent quiescent, intermediate, and fully activated states, respectively. (**A**) Relative mRNA expression of *glutaminase 1* (*GLS1*), *serine hydroxymethyltransferase 2* (*SHMT2*), and *aldehyde dehydrogenase 18 family member A1* (*ALDH18A1*) in human qHSCs versus aHSCs. (**B**) Gene set enrichment analysis (GSEA) plot for the “WP_METABOLIC_PATHWAYS_OF_FIBROBLASTS” gene set, comparing transcriptional profiles of human qHSCs and aHSCs. (**C**) Representative bright-field images (scale bar: 100 μm) and Western blot analysis with corresponding quantification of collagen type I, alpha-smooth muscle actin (α-SMA), GLS1, SHMT2, and pyrroline-5-carboxylate synthase (P5CS) protein levels in primary rat HSCs at different activation states (Day 1, 3, and 7) (*n* = 6 per group). Data are presented as mean ± SD. For (**A**), statistical significance was determined using a two-tailed Student’s *t*-test. For (**C**), a one-way ANOVA followed by Tukey’s post hoc test was used. * *p* < 0.05, ** *p* < 0.01, *** *p* < 0.001.

To determine whether HSC activation is associated with metabolic shifts, we first analyzed public transcriptomic data [29]. The expression of key genes involved in glutaminolysis (*GLS1*), de novo glycine synthesis (*SHMT2*), and proline synthesis (*ALDH18A1*) was significantly elevated in aHSC compared to their quiescent counterparts (GSE67664, Figure 1A). To confirm this trend at a broader pathway level, we performed GSEA on an independent dataset (GSE68001) [30]. This analysis revealed that the METABOLIC PATHWAYS OF FIBROBLASTS gene set was significantly enriched in aHSCs relative to qHSC. (Figure 1B), reinforcing the link between HSC activation and metabolic alterations. We next sought to validate these transcriptomic findings at the protein level using an in vitro model of spontaneous HSC activation. Primary rat HSCs were cultured for up to seven days, representing a transition from a quiescent state (day 1) to an intermediate (day 3) and then a fully activated state (day 7). Western blot analysis demonstrated that the protein levels of GLS1, SHMT2, and P5CS increased in a time-dependent manner. Importantly, this increase closely paralleled the upregulation of established activation markers, including collagen type I and α-SMA (Figure 1C). Collectively, these transcriptomic and protein-level data indicate that the activation of HSCs is characterized by a significant upregulation of key amino acid metabolic pathways.

### 3.2. Pirfenidone Suppresses HSC Activation Markers Post-Transcriptionally and Inhibits Cell Proliferation

**Figure 2:**
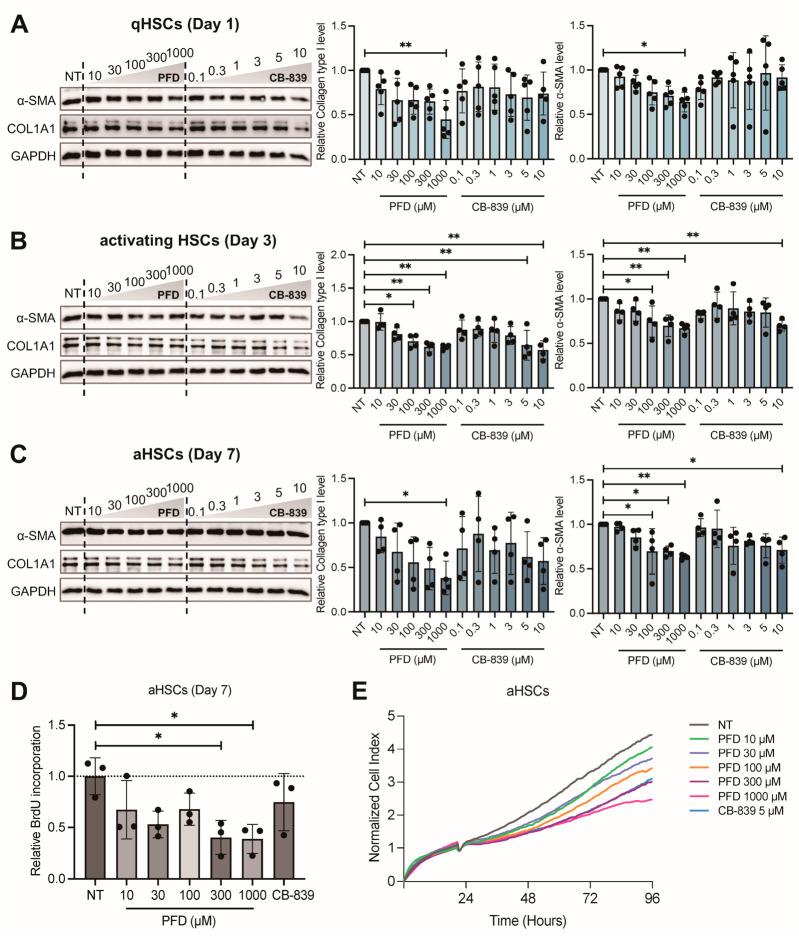
Pirfenidone suppresses activation markers and inhibits the proliferation of aHSCs. Primary rat HSCs were treated with day 1 (qHSC), day 3 (activating), and day 7 (aHSC) post-isolation. The cells were exposed to a dose range of pirfenidone (PFD, 10–1000 μM) or the glutaminase 1 (GLS1) inhibitor CB-839 (0.1–10 μM) for 72 h. (**A**–**C**) Western blot and quantitative analysis of the activation markers collagen type I and alpha-smooth muscle actin (α-SMA) in qHSCs (Day 1; *n* = 5), activating HSCs (Day 3; *n* = 4) and aHSCs (Day 7; *n* = 4). (**D**) Quantification of aHSC proliferation by bromodeoxyuridine (BrdU) incorporation assay following treatment with PFD or CB-839 (5 μM) (*n* = 3 per group). (**E**) Real-time monitoring of aHSC proliferation using the xCELLigence Real-Time Cell Analysis (RTCA) system, presented as a Normalized Cell Index. Data are presented as mean ± SD. The statistical significance of differences was analyzed by one-way ANOVA followed by Tukey’s post hoc test. * *p* < 0.05, ** *p* < 0.01.

**Figure 3:**
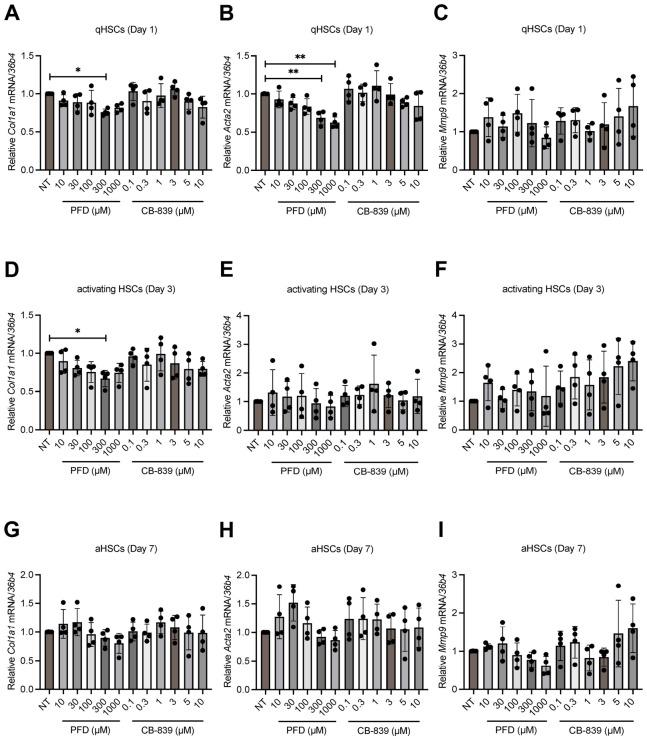
Pirfenidone minimally affects the transcription of key activation markers. Primary rat HSCs at quiescent (Day 1), intermediate (Day 3), and fully activated (Day 7) states were treated with the indicated doses of pirfenidone (PFD) or CB-839 for 72 h (*n* = 4 per group). (**A**–**C**) Relative mRNA levels of *collagen type I* (*Col1a1*), *alpha-smooth muscle actin* (*Acta2*), and *matrix metallopeptidase 9* (*Mmp9*) in qHSCs. (**D**–**F**) Relative mRNA levels of the same markers in activating HSCs. (**G**–**I**) Relative mRNA levels in aHSCs. Data are presented as mean ± SD. The statistical significance of differences was analyzed by one-way ANOVA followed by Tukey’s post hoc test. * *p* < 0.05, ** *p* < 0.01.

To evaluate the anti-fibrotic potential of PFD, we treated primary rat HSCs at quiescent, activating, and fully activated stages with increasing concentrations of PFD. To determine the specific contribution of glutaminolysis inhibition to these effects, the GLS1 inhibitor CB-839 was included as a reference compound. PFD dose-dependently reduced the protein levels of activation markers across all stages. In qHSCs, 1000 μM PFD reduced collagen type I and α-SMA levels by 55.3% and 36.1%, respectively, whereas CB-839 had no significant effect (Figure 2A). In activating HSCs, PFD was effective from 100 μM, with the 1000 μM dose downregulating collagen type I by 38.4% and α-SMA by 33%. CB-839 also exhibited an inhibitory effect at this stage (Figure 2B). Notably, in aHSCs, PFD treatment decreased both collagen type I and α-SMA by 61.7% and 36.7%. In contrast, CB-839 only reduced α-SMA expression (Figure 2C). In addition to suppressing activating markers, we assessed the impact of PFD on aHSC proliferation using BrdU incorporation and xCELLigence RTCA. Both assays demonstrated that 1000 μM PFD reduced aHSC proliferation compared to non-treated controls. While CB-839 showed a trend towards reduced proliferation, the effect did not reach statistical significance (Figure 2D,E). To investigate whether the reduction in activation markers occurred at the transcriptional level, we performed qPCR analysis. Surprisingly, neither PFD nor CB-839 treatment substantially altered the mRNA levels of *Col1a1*, *Acta2*, or matrix metallopeptidase 9 (*Mmp9*) in HSCs at any stage of activation (Figure 3A–I). These results suggest that PFD suppresses the expression of key activation markers primarily through a post-transcriptional mechanism, distinguishing its action from direct transcriptional repression.

### 3.3. Pirfenidone Attenuates Pro-Fibrotic Metabolism by Downregulating Key Amino Acid Metabolic Enzymes

**Figure 4:**
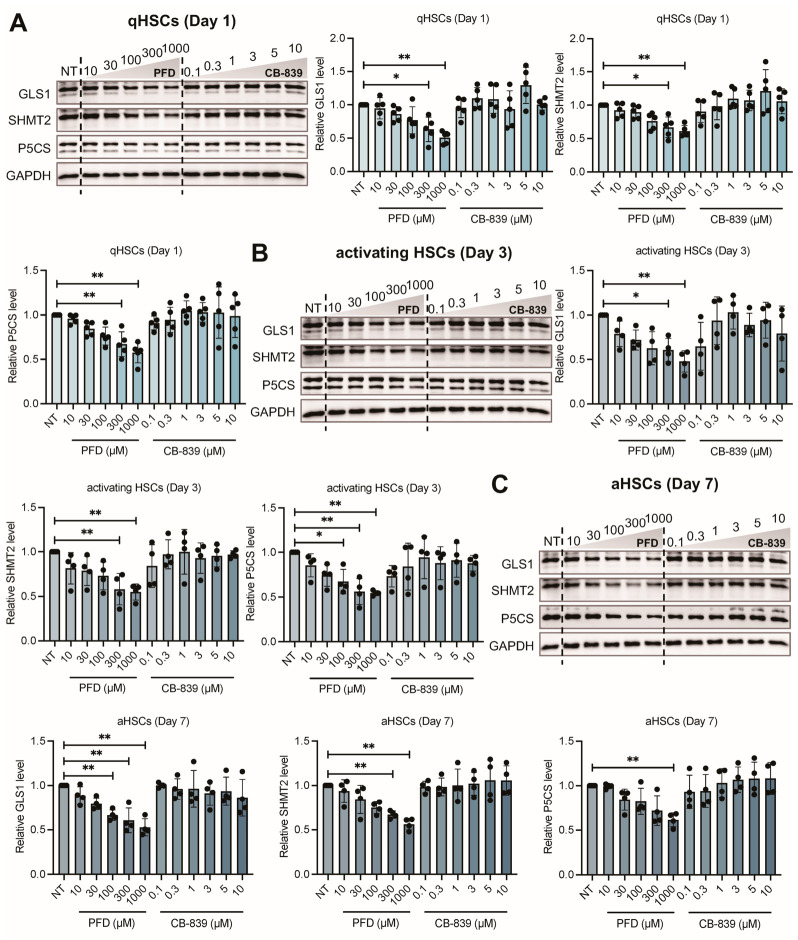
Pirfenidone treatment reduces protein levels of key metabolic enzymes in HSCs. Primary rat HSCs at quiescent (qHSCs, Day 1), intermediate (Day 3), and fully activated (aHSCs, Day 7) states were treated with pirfenidone (PFD, 10–1000 μM) or CB-839 for 72 h. (**A**–**C**) Western blot and corresponding quantitative analysis of glutaminase 1 (GLS1), serine hydroxymethyltransferase 2 (SHMT2), and pyrroline-5-carboxylate synthase (P5CS) protein levels in qHSCs (*n* = 5), intermediate activating HSCs (*n* = 4), and aHSCs (*n* = 4). Data are presented as mean ± SD. The statistical significance of differences was analyzed by one-way ANOVA followed by Tukey’s post hoc test. * *p* < 0.05, ** *p* < 0.01.

Given that HSC activation is characterized by metabolic reprogramming, we hypothesized that PFD exerts its anti-fibrotic effects by targeting this process. We therefore measured the protein expression of key metabolic enzymes in HSCs at different activation stages following PFD treatment. PFD induced a concentration-dependent downregulation of GLS1, SHMT2, and P5CS, which are critical enzymes for glutamine, glycine, and proline metabolism, respectively. This effect was observed consistently across all stages. Specifically, in qHSCs, a 1000 μM PFD treatment reduced GLS1, SHMT2, and P5CS levels by 49.1%, 39.1%, and 42.5% (Figure 4A). A similar magnitude of reduction was observed in activating HSCs, with a decrease of 52.1%, 45.1%, and 45.6% (Figure 4B), and fully activated HSCs, with a decrease of 47.2%, 43.8%, and 38.5% (Figure 4C). In contrast, and consistent with its function as a direct enzymatic inhibitor, CB-839 did not reduce the protein expression level of its target, GLS1. This highlights a difference in the mechanism of action between PFD and a targeted metabolic inhibitor. Taken together, these data demonstrate that PFD’s anti-fibrotic activity is associated with a marked suppression of the protein abundance of key metabolic enzymes. This suggests PFD works, at least in part, by dismantling the metabolic infrastructure required to fuel HSC activation and matrix production.

### 3.4. Pirfenidone Inhibits ATF4 Nuclear Accumulation and Impairs Mitochondrial Respiratory Capacity

**Figure 5:**
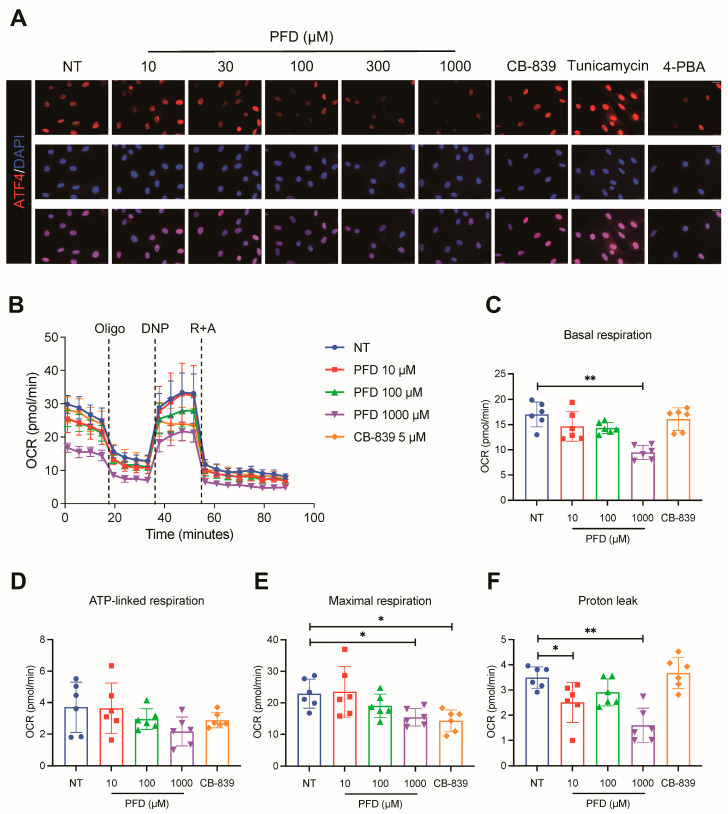
Pirfenidone prevents ATF4 nuclear accumulation and suppresses mitochondrial respiration in aHSCs. aHSCs were treated with pirfenidone (PFD, 10–1000 μM) or the glutaminase 1 (GLS1) inhibitor CB-839 (5 μM) for 72 h. For activating transcription factor 4 (ATF4) analysis, tunicamycin and 4-phenylbutyrate (4-PBA) were administered as positive and negative controls, respectively, during the final 12 h of treatment. (**A**) Representative immunofluorescence images of ATF4 localization (scale bar: 25 μm). (**B**–**F**) Quantification of mitochondrial respiration parameters in treated aHSCs, measured using a Seahorse XF96 Analyzer. The analysis shows (**C**) basal respiration, (**D**) ATP-linked respiration, (**E**) maximal respiration, and (**F**) proton leak, derived from the oxygen consumption rate (OCR) profile after sequential injections of oligomycin (Oligo), dinitrophenol (DNP), and a mixture of rotenone and antimycin A (R + A). Data are presented as mean ± SD. The statistical significance of differences was analyzed by one-way ANOVA followed by Tukey’s post hoc test. * *p* < 0.05, ** *p* < 0.01.

To identify the upstream regulator responsible for the PFD-mediated downregulation of metabolic enzymes, we investigated the transcription factor ATF4, a known master regulator of amino acid metabolism. Immunofluorescence analysis in aHSCs revealed that PFD treatment dose-dependently inhibited the nuclear accumulation of ATF4. This effect was demonstrated by a reduction in the colocalization of ATF4 with the DAPI-stained nucleus, similar to the effect of the endoplasmic reticulum (ER) stress inhibitor 4-PBA and opposite to the inducer tunicamycin (Figure 5A). This finding suggests that PFD interferes with the ATF4 signaling axis upstream of metabolic enzyme expression. Given that ATF4-regulated metabolic pathways are central to mitochondrial function, we next evaluated the bioenergetic profile of aHSCs using a Seahorse XF metabolic flux analyzer. PFD treatment (1000 μM) impaired mitochondrial function. Most notably, it caused a reduction in the maximal respiratory capacity of aHSCs, indicating that the cells lost their ability to produce energy in response to increased demand. PFD also decreased basal respiration and proton leak. In contrast, the GLS1 inhibitor CB-839 only affected maximal respiration and did not alter other parameters (Figure 5B–F). Crucially, neither PFD nor CB-839 altered adenosine triphosphate (ATP)-linked respiration, suggesting the primary effect of PFD is not a reduction in baseline ATP production but rather a critical loss of metabolic flexibility. Collectively, these findings demonstrate that PFD inhibits the nuclear accumulation of the master metabolic regulator ATF4, an action coupled with an impaired mitochondrial respiratory capacity that would limit the energetic potential required to sustain HSC activation.

### 3.5. Pirfenidone Sensitizes aHSCs to Ferroptosis by Disrupting Glutathione Homeostasis

**Figure 6:**
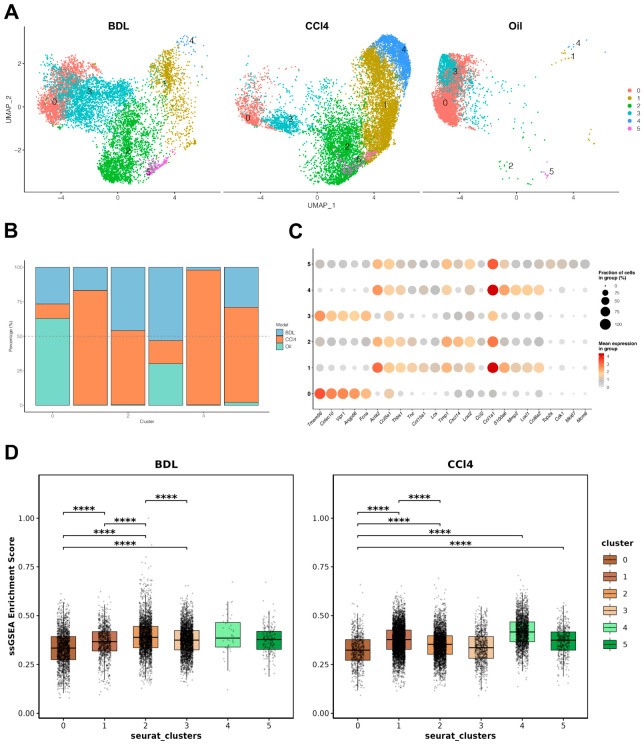
Activated HSCs acquire ferroptosis resistance during liver injury. A single-cell RNA-sequencing dataset (GSE171904) was analyzed to identify murine HSC subpopulations from mice treated with a vehicle control (Oil), carbon tetrachloride (CCl4), or subjected to bile duct ligation (BDL). (**A**) UMAP visualization of HSC cluster compositions across the three experimental conditions. (**B**) Stacked bar plot showing the proportional representation of each HSC cluster in each condition. (**C**) Dot plot visualizing the expression of representative cell phenotype markers. The dot size corresponds to the percentage of cells in the cluster expressing the gene, and the color intensity reflects the average expression level. (**D**) Single-sample gene set enrichment analysis (ssGSEA) scores for the “GOBP_NEGATIVE_REGULATION_OF_FERROPTOSIS” gene set across HSC clusters from the CCl4 and BDL models. **** *p* < 0.0001.

Given that aHSCs employ metabolic adaptations to resist cell death, we first sought to confirm this phenotype using a public single-cell RNA sequencing dataset from murine liver fibrosis models [31]. Unbiased clustering of HSCs from control, BDL, and CCl4-treated mice identified six distinct subpopulations (Figure 6A,B). Cluster 0 represented qHSCs, while clusters 1 through 5 displayed feature of activation, including early activation (cluster 1, high *Acta2*), matrix production (cluster 4, high *Col1a1*), and proliferation (cluster 5, high *Mki67*) (Figure 6C). Notably, the matrix-producing cluster 4 constituted a minor fraction of cells from the BDL model, an observation consistent with the role of portal fibroblasts as the primary collagen producers in that specific injury context [31]. Critically, gene set enrichment analysis revealed that all aHSC clusters (1–5) showed significant enrichment of pathways associated with the negative regulation of ferroptosis compared to the quiescent cluster 0. This indicates that HSCs acquire ferroptosis resistance as a survival mechanism during activation (Figure 6D). To further characterize ferroptosis resistance mechanisms in aHSCs, we examined the expression patterns of key ferroptosis regulators across HSC subpopulations (Figure S1A). *Gpx4* converts lipid hydroperoxides to non-toxic lipid alcohols through GSH-dependent reduction, *Fth1* encodes the iron storage protein ferritin that sequesters free iron to limit its availability for lipid peroxidation, while *Aifm2*, also known as ferroptosis suppressor protein 1, catalyzes the regeneration of coenzyme Q10 to trap lipid peroxyl radicals, and *Gch1* drives the synthesis of the antioxidant tetrahydrobiopterin that suppresses ferroptosis by preventing depletion of polyunsaturated phospholipids. Among these genes, *Gpx4* and *Fth1* displayed higher mean expression levels and broader cellular distribution across all HSC clusters, with over 60–100% of cells showing detectable expression. In contrast, *Aifm2* and *Gch1* exhibited relatively sparse expression patterns, with fewer than 15% of cells expressing these genes in most clusters (Figure S1B). This expression pattern suggests that the GSH-GPX4 axis likely serves as the predominant ferroptosis resistance mechanism during HSC activation, while alternative pathways mediated by *Aifm2* and *Gch1* may play supplementary roles in specific HSC subpopulations.

**Figure 7:**
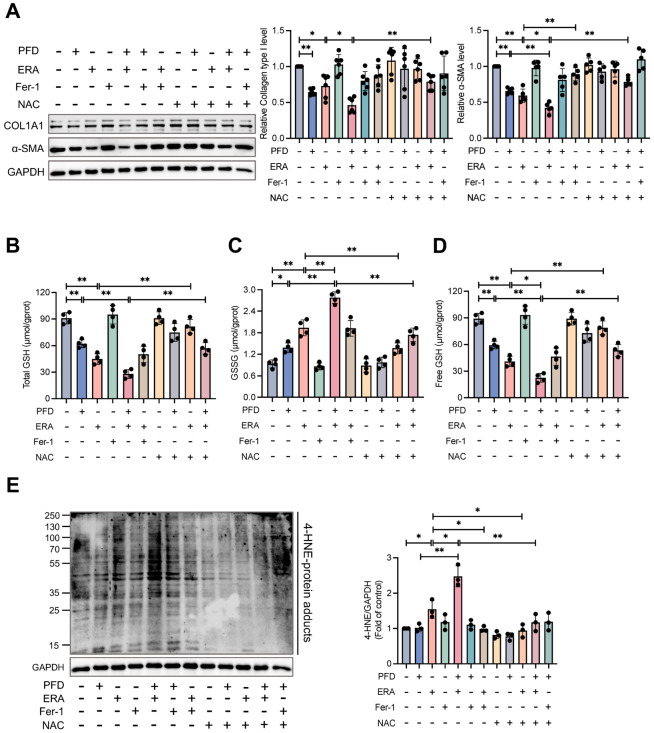
Pirfenidone sensitizes aHSCs to ferroptosis by disrupting glutathione homeostasis and promoting lipid peroxidation. aHSCs were treated with pirfenidone (PFD) for 72 h. During the final 24 h of incubation, cells were co-treated with either the ferroptosis inducer erastin (ERA, 10 μM), the ferroptosis inhibitor ferrostatin-1 (Fer-1, 1 μM), or the antioxidant N-acetylcysteine (NAC, 2 mM). (**A**) Western blot and quantitative analysis of collagen type I (*n* = 6) and α-SMA (*n* = 5) protein levels. (**B**–**D**) Quantification of total glutathione (GSH), oxidized glutathione (GSSG), and the free GSH pool. (**E**) Protein expression levels of the lipid peroxidation marker 4-hydroxynonenal (4-HNE), presented as fold change relative to untreated controls (*n* = 3 per group). Data are presented as mean ± SD. The statistical significance of differences was analyzed by one-way ANOVA followed by Tukey’s post hoc test. * *p* < 0.05, ** *p* < 0.01.

We therefore hypothesized that PFD’s metabolic disruption could reverse this resistance and sensitize aHSCs to ferroptosis. The combination of PFD and ferroptosis inducer erastin (ERA) synergistically suppressed HSC activation, reducing collagen type I and α-SMA protein levels by 53.7% and 57.6%, respectively. This potent anti-fibrotic effect was abrogated by the ferroptosis inhibitors Fer-1 and NAC, confirming that the synergy was mediated through ferroptotic cell death (Figure 7A). To elucidate the mechanism of this sensitization, we investigated GSH metabolism, the cell’s primary defense against ferroptosis. PFD and ERA target two distinct but complementary arms of GSH synthesis. PFD treatment alone reduced total and free GSH levels by inhibiting the production of the precursors glutamate and glycine. ERA inhibits the import of the third precursor, cystine. As hypothesized, the combination of PFD and ERA caused a collapse in GSH homeostasis, reducing total and free GSH by 69.1% and 74.7% and tripling the oxidized GSSG content. This effect was partially reversed by NAC, which replenishes the cysteine pool for GSH synthesis (Figure 7B–D).

**Figure 8:**
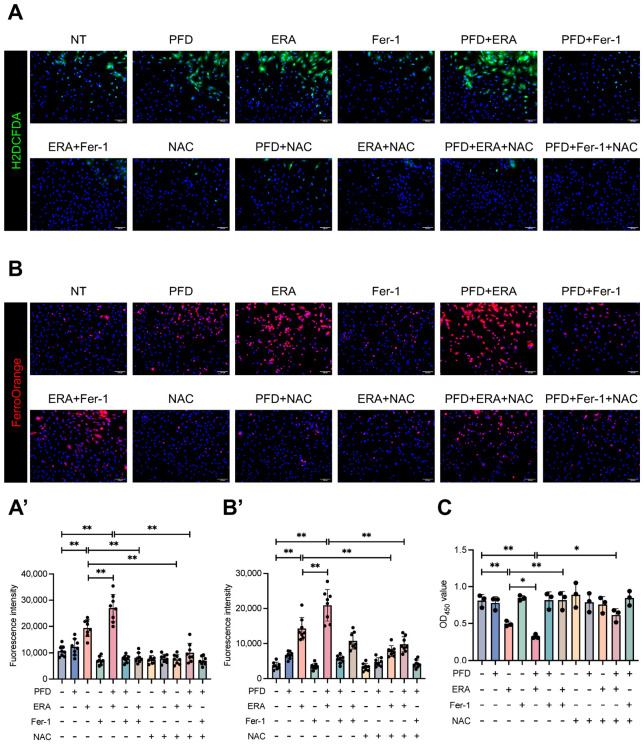
Pirfenidone potentiates erastin-induced ROS production, labile iron accumulation, and cell death in aHSCs. aHSCs were treated as described in Figure 7. (**A**) Representative fluorescence images and corresponding intensity quantification of intracellular reactive oxygen species (ROS) using 2′,7′-dichlorodihydrofluorescein diacetate (H2DCFDA) staining (scale bar: 100 μm). (**A’**) Data were quantified from 8 random fields per group. (**B**) Representative fluorescence images and corresponding intensity quantification of the intracellular labile iron pool using FerroOrange staining (scale bar: 100 μm). (**B’**) Data were quantified from 8 random fields per group. (**C**) Cell viability was assessed by Cell Counting Kit-8 (CCK-8) assay (*n* = 3 per group). Data are presented as mean ± SD. The statistical significance of differences was analyzed by one-way ANOVA followed by Tukey’s post hoc test. * *p* < 0.05, ** *p* < 0.01.

The depletion of GSH impairs the cell’s ability to neutralize lipid peroxides, the executioners of ferroptosis [17]. Accordingly, combined PFD and ERA treatment led to a massive accumulation of the lipid peroxidation marker 4-hydroxynonenal (4-HNE) and an increase in intracellular ROS, effects that were reversed by ferroptosis inhibitors (Figure 7E and Figure 8A). A terminal event in ferroptosis is the iron-dependent catalysis of these lipid peroxides. We observed that PFD and ERA co-treatment increased the labile iron pool (LIP). Notably, the radical scavenger Fer-1, which acts downstream of iron, failed to reverse this LIP increase. In contrast, NAC, by restoring GSH synthesis, prevented the upstream oxidative stress that drives iron dysregulation and successfully normalized the LIP (Figure 8B). Finally, viability assays confirmed this synergy, showing that PFD co-treatment significantly enhanced ERA-induced cell death by 34.8%, an effect far greater than simple additive toxicity (Figure 8C). Collectively, these results demonstrate that PFD remodels HSC metabolism to create a state of profound vulnerability to ferroptosis. It sensitizes aHSCs to ferroptosis by crippling their intrinsic antioxidant defenses, setting the stage for a synergistic and lethal collapse when combined with a direct ferroptosis inducer.

## 4. Discussion

**Figure 9:**
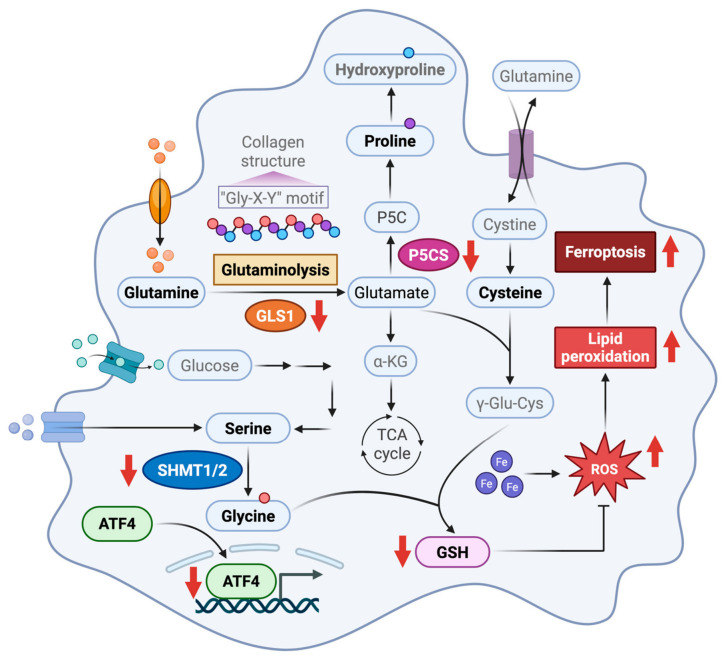
Schematic of the mechanism by which pirfenidone remodels metabolism and sensitizes hepatic stellate cells to ferroptosis. Pirfenidone inhibits the nuclear accumulation of the metabolic transcription factor ATF4. This suppression downregulates the expression of key metabolic enzymes including glutaminase 1 (GLS1), serine hydroxymethyltransferase 2 (SHMT2), and pyrroline-5-carboxylate synthase (P5CS). This coordinated downregulation has two consequences for HSC fate. First, it impairs glutaminolysis, de novo glycine synthesis, and proline synthesis, thereby limiting the availability of precursors for collagen production and inhibiting HSC activation. Second, the reduced supply of glutamate and glycine, which are essential substrates for glutathione (GSH) synthesis, leads to GSH depletion. This depletion sensitizes the cells to lipid peroxidation and subsequent ferroptosis. Red arrows indicate points of downregulation (↓) or upregulation (↑) within the metabolic network following pirfenidone treatment. This figure was created in BioRender. Li, J. (2026), https://BioRender.com/35yqyyj (accessed on 20 March 2026).

Liver fibrosis, a consequence of chronic liver disease, is driven by the activation of HSCs and the subsequent excessive deposition of ECM. This pathological transformation of HSCs into fibrogenic myofibroblasts is a central event in disease progression [32]. Although PFD has shown therapeutic promise against liver fibrosis, its underlying molecular mechanisms remain incompletely understood [33,34,35,36]. Our study addressed this knowledge gap by identifying a distinct metabolic signature of HSC activation, characterized by the upregulation of glutamine catabolism, serine/glycine synthesis, and proline biosynthesis. We then demonstrated that PFD counteracted this pro-fibrotic state. Our findings indicated that PFD inhibited the nuclear accumulation of the key metabolic transcription factor ATF4. This upstream event was associated with two significant consequences. First, it limited the availability of precursors for collagen synthesis. Second, it depleted the substrates required for GSH synthesis (Figure 9). Based on these results, we propose a novel, dual-pronged mechanism for PFD’s antifibrotic action. PFD not only suppresses the metabolic engine essential for HSC action but also sensitizes these cells to ferroptosis by dismantling their antioxidant defense.

Metabolic reprogramming is a recognized hallmark of cellular activation, and our findings specifically implicate amino acid metabolism as a cornerstone of the HSC activated phenotype. This aligns with and expands upon existing literature. The critical role of glutaminolysis in driving fibrosis is well-documented [37,38,39]. In vivo studies have shown that glutamine metabolism in HSCs is a feature of active fibrogenesis [8,9]. Our work reinforced this by demonstrating that PFD-mediated suppression of the key enzyme GLS1 correlated with reduced HSC activation. Furthermore, our results highlighted the importance of one-carbon metabolism and proline synthesis. The enzyme SHMT2 and P5CS, which are central to these pathways, provide the glycine and proline backbones that constitute over half of collagen’s amino acid content. Previous reports have shown that SHMT2 is elevated in HSCs from fibrotic livers and that proline synthesis is a rate-limiting step for collagen production [13,40]. By demonstrating that key enzymes (GLS1, SHMT2, and P5CS) from all three of these interconnected pathways were upregulated during HSC activation, our study presented a more holistic view of the metabolic dependency of fibrotic cells. This suggests that targeting this metabolic network offers a promising therapeutic strategy to restore HSC quiescence. However, a limitation of our study is the reliance on protein expression data. Future work employing stable isotope tracing would be necessary to directly quantify metabolic flux through these pathways and definitively confirm their functional activity.

PFD is an established antifibrotic agent known to modulate signaling pathways such as transforming growth factor-beta (TGF-β), yet its effects on cellular metabolism have been less explored [41,42]. Our research provides insight by demonstrating that PFD acts as a potent regulator of amino acid metabolism in HSCs. We observed that PFD effectively suppressed cell proliferation and dose-dependently downregulated protein levels of activation markers. Notably, this inhibitory effect was more pronounced than that of the specific GLS1 inhibitor CB-839. A key finding was that neither PFD nor CB-839 significantly altered the mRNA levels of these activation markers, suggesting a post-transcriptional or translational mode of regulation. Subsequent analysis revealed that PFD’s impact extends beyond glutaminolysis. It also suppressed the protein expression of SHMT2 and P5CS, the key enzyme for glycine and proline synthesis, respectively. This coordinated downregulation of three distinct but synergistic metabolic pathways explains PFD’s superior efficacy compared to a single-pathway inhibitor. These findings suggest that PFD cripples the fibrogenic program by simultaneously cutting off the energy supply, the building blocks for collagen, and the components for antioxidant defense, all of which are required for sustained HSC activation.

The coordinated downregulation of GLS1, SHMT2, and P5CS by PFD points toward a common upstream regulator. Our study identified ATF4 as a compelling candidate. We discovered that PFD inhibited the nuclear accumulation of ATF4, preventing it from executing its transcriptional program. This finding provides a unifying mechanism for PFD’s broad metabolic effects. The link between ATF4 and these metabolic pathways is supported by existing evidence. ATF4 is a known downstream effector of the pro-fibrotic TGF-β pathway and directly regulates the expression of serine/glycine synthesis enzymes, including SHMT2 [43,44]. Furthermore, P5CS is a well-established ATF4 target gene, while increased ATF4 activity has also been reported to elevate GLS1 expression, suggesting it is at least indirectly regulated by this transcription factor [45,46,47]. Our data therefore position ATF4 as a critical node where PFD intersects the non-canonical TGF-β signaling cascade to reprogram HSC metabolism. The importance of ATF4 in HSC biology is further underscored by recent studies demonstrating that HSC-specific ATF4 depletion suppresses liver fibrosis in vivo, that pharmacological inhibition of ATF4 translation effectively mitigates fibrosis, and that ATF4 promotes fibroblast activation through the tribbles pseudokinase 3-mediated mitochondrial stress pathway [48]. A key mechanistic question arising from our findings is how PFD reduces ATF4 nuclear accumulation. ATF4 is primarily regulated at the translational level through the integrated stress response. In this pathway, stress-activated eukaryotic initiation factor-2α (eIF2α) kinases, particularly protein kinase R-like endoplasmic reticulum kinase (PERK), phosphorylate eIF2α, which in turn enhances ATF4 mRNA translation via upstream open reading frames. Notably, ATF4 contains an intrinsic nuclear localization signal that mediates its constitutive nuclear import upon synthesis [49], and the protein is inherently unstable with a half-life of only 30–60 min due to rapid SCF^βTrCP^-mediated proteasomal degradation [50]. These properties indicate that nuclear ATF4 abundance is determined predominantly by the rate of translation rather than by a separately regulated nuclear import step. Importantly, the PERK–eIF2α–ATF4 signaling pathway has been shown to be activated during the progression of liver fibrosis [51], establishing this pathway as a disease-relevant regulatory axis. Our observation that PFD recapitulated the effect of the ER stress inhibitor 4-PBA on ATF4 nuclear localization, while opposing the effect of the ER stress inducer tunicamycin, provides a critical experimental clue. Given PFD’s well-documented antioxidant properties and its capacity to attenuate ER stress, we propose that PFD reduces ATF4 nuclear accumulation primarily by alleviating cellular stress signals that drive PERK–eIF2α activation, thereby suppressing ATF4 translational upregulation. While our evidence points towards the PERK pathway, other signaling inputs could also contribute to this effect. For instance, PFD’s known modulation of TGF-β signaling might influence ATF4 translation via the mammalian target of rapamycin complex 1 axis, and its impact on cellular metabolism could potentially engage the amino acid-sensing general control nonderepressible 2 pathway [44,52]. It should be noted that our assessment of ATF4 nuclear localization was based on immunofluorescence, and future studies employing subcellular fractionation with Western blot analysis, together with direct measurement of eIF2α phosphorylation status and PERK activation, will be essential to validate this proposed mechanism. Importantly, our data indicate that the inhibition of ATF4 nuclear accumulation and the sensitization of aHSCs to ferroptosis are not independent, parallel effects of PFD, but rather represent the possible upstream cause and downstream consequence within the same mechanistic cascade. ATF4 inhibition leads to the coordinated downregulation of GLS1 and SHMT2, which reduces the intracellular availability of glutamate and glycine, two of the three essential precursors for GSH biosynthesis. This ATF4-dependent GSH depletion creates the metabolic vulnerability that, when compounded by erastin-mediated blockade of cystine import, precipitates the synergistic collapse of antioxidant defense and ferroptotic cell death. Thus, ATF4 nuclear accumulation inhibition emerges as the pivotal upstream event that initiates both arms of PFD’s dual anti-fibrotic mechanism: suppressing collagen synthesis and sensitizing aHSCs to ferroptosis. Future rescue experiments employing ATF4 overexpression will be essential to definitively establish this causal hierarchy by determining whether restoring ATF4 activity can reverse PFD-induced GSH depletion and abrogate ferroptosis sensitization. This redefines PFD’s mechanism, shifting its perception from a broad-spectrum inhibitor to a more targeted metabolic modulator acting on the ATF4 axis. This axis consequently emerges as a potential target for future antifibrotic drug development [53]. The inhibition of these ATF4-dependent enzymes also explains the observed impairment in mitochondrial function. Notably, the specific bioenergetic pattern induced by PFD, a reduction in maximal respiratory capacity with preserved ATP-linked respiration, indicates a loss of metabolic flexibility rather than an acute bioenergetic crisis. This suggests that PFD-treated cells can meet basal energy demands but have a critically diminished capacity to respond to metabolic stress. This phenotype can be mechanistically attributed to the dual suppression of GLS1 and SHMT2. The reduction in maximal respiration is consistent with substrate limitation from the GLS1-glutaminolysis axis, as specific GLS1 inhibition with CB-839 is known to reduce TCA cycle intermediates and similarly impairs maximal respiratory capacity [54]. However, PFD elicited a broader bioenergetic impairment than the GLS1-specific inhibitor, also affecting basal respiration. This additional effect is logically explained by the concurrent suppression of SHMT2. SHMT2-derived one-carbon units are essential for the translation of mitochondrial-encoded respiratory subunits, and reduced SHMT2 expression has been shown to impair basal respiration and overall oxidative capacity [55,56]. Therefore, the comprehensive mitochondrial phenotype seen with PFD appears to be a composite effect of substrate limitation and impaired respiratory chain assembly, stemming from the simultaneous inhibition of both GLS1 and SHMT2. We acknowledge, however, that this model is based on protein expression and bioenergetic profiling. Future studies employing stable isotope tracing with ^13^C-glutamine and ^13^C-serine will be essential to directly validate this proposed substrate-supply mechanism and definitively establish the causal hierarchy.

It is important to acknowledge that our study, by design, focused on a specific metabolic axis, whereas PFD is a pleiotropic agent with effects extending beyond the pathways investigated here. In HSCs, PFD is known to modulate canonical pro-fibrotic signaling, including the TGF-β/Smad and Wnt pathways, and to regulate protein S-glutathionylation via glutaredoxin-1, which also impacts Smad activity [33,41,42,57]. Furthermore, PFD exerts effects on other liver cells that were not captured by our isolated primary HSC culture system. For instance, it can protect hepatocytes from apoptosis and modulate macrophage polarization towards a restorative phenotype, both of which would indirectly suppress HSC activation in a context [58,59]. The ferroptosis inducer erastin also has non-canonical mitochondrial targets, including voltage-dependent anion-selective channel 2/3, which could contribute to the oxidative stress observed in our experiments [60]. Therefore, while our findings provide a coherent model linking PFD’s action to the ATF4-amino acid-GSH axis, its overall therapeutic efficacy likely results from the convergence of these direct metabolic effects on HSCs with its broader actions on multiple signaling networks and cell types.

A particularly significant implication of our work is the discovery that PFD sensitizes aHSCs to ferroptosis. While inducing ferroptosis is an emerging antifibrotic strategy, our single-cell analysis revealed that aHSCs were inherently resistant to this form of cell death. We demonstrated a synergy between PFD and the ferroptosis inducer ERA. This effect is rooted in the central role of the antioxidant GSH. GSH synthesis requires glutamate, cysteine, and glycine. ERA blocks cysteine import by inhibiting the system Xc^−^ transporter [61]. Our data showed that PFD complemented this action by reducing the other two precursors. The PFD-mediated downregulation of GLS1 and SHMT2 reduced the intracellular pools of glutamate and glycine, respectively. This “two-hit” mechanism leads to a collapse of the GSH system, which neither agent can achieve alone. Our experiments confirmed that the combination treatment markedly depleted GSH, increased lipid peroxidation, and elevated intracellular labile iron, all hallmarks of ferroptosis. This finding resolves an apparent paradox in the previous study regarding a pro-ferroptotic role for glutaminolysis mediated specifically by GLS2, where its activity fuels mitochondrial ROS production. This was substantiated by the observation that the GLS1-specific inhibitor BPTES failed to impact serum-dependent ferroptosis [62]. In contrast, our work reveals a protective role for the GLS1-dependent pathway, which is essential for supplying the glutamate required for synthesizing the antioxidant GSH. The combination of PFD and ERA led to the most profound depletion of both total and free GSH, accompanied by a significant increase in GSSG, indicating severe oxidative stress. Concurrently, we observed an accumulation of key ferroptosis markers, including the lipid peroxidation product 4-HNE and LIP. Crucially, these effects, along with the synergistic reduction in cell viability, were reversed by the ferroptosis inhibitors Fer-1 and NAC, confirming the ferroptotic nature of the cell death and the central role of GSH collapse. These findings position PFD not merely as a broad-spectrum inhibitor, but as a specific metabolic modulator that strategically dismantles the ferroptosis defense network in aHSCs. This shifts the therapeutic paradigm from “single-agent induction” to a more sophisticated “combination-sensitization” strategy, holding promise for antifibrotic therapies. A key limitation of our study is that these synergistic effects were demonstrated exclusively in vitro, and their validation in preclinical animal models is the most critical next step. While erastin served as the canonical tool compound to establish our mechanistic proof-of-concept, its poor metabolic stability precludes direct in vivo application. For translational development, clinically relevant alternatives are necessary. Several FDA-approved drugs represent promising candidates, including sulfasalazine, a well-characterized system Xc^−^ inhibitor [63]. More notably, the multikinase inhibitor sorafenib, already used for hepatocellular carcinoma, has been shown to trigger ferroptosis selectively in aHSCs and attenuate fibrosis in a murine model, providing a strong precedent for its use in a combination strategy [64]. However, the translation of any ferroptosis-based therapy for liver fibrosis must confront the “double-edged sword” nature of this cell death pathway. While inducing ferroptosis in aHSCs is an emerging antifibrotic strategy [65,66], hepatocyte ferroptosis is a known driver of liver injury and inflammation, particularly in metabolic liver disease [67,68]. This concern is amplified by findings that chronic inhibition of system Xc^−^ can paradoxically exacerbate hepatocyte injury, as hepatocytes also upregulate this transporter for survival under sustained stress [69]. Of particular relevance to our findings, ATF4 was recently shown to protect normal hepatocytes from ferroptosis by maintaining GSH production [70]. Since our data identify PFD as an inhibitor of ATF4 nuclear accumulation, the potential impact on hepatocyte viability warrants careful in vivo evaluation. Nonetheless, several lines of evidence suggest that PFD-mediated sensitization may be preferentially directed toward aHSCs, potentially offering a wider therapeutic window than direct system Xc^−^ inhibitors. First, PFD’s metabolic targets, including GLS1 and SHMT2, are specifically upregulated during HSC activation, creating a vulnerability that is not shared by hepatocytes [9,71]. Second, PFD’s mechanism of depleting GSH precursors is distinct from direct cystine import blockade. This alternative mode of sensitization may enable synergistic efficacy with lower, non-toxic doses of a clinical inducer like sorafenib. Third, PFD itself has demonstrated hepatoprotective properties and an acceptable safety profile in clinical trials with liver disease patients [27,72]. Future preclinical studies should therefore test PFD in combination with sorafenib in established fibrosis models. Such studies must incorporate rigorous safety assessments, including liver function tests and cell-type-specific co-localization of ferroptosis markers with HSC and hepatocyte identifiers, to definitively evaluate both the therapeutic efficacy and the safety of this combination strategy.

An important consideration for the clinical translation of our findings is the use of PFD at concentrations (100–1000 μM) that are higher than the typical peak plasma levels (Cmax, ~43–80 μM) observed in patients. However, several pharmacological factors argue that the drug exposure at the target tissue site is well within this experimental range. First, as an orally administered drug, PFD undergoes extensive first-pass hepatic uptake, leading to preferential accumulation in the liver. Pharmacokinetic studies have demonstrated that the liver-to-plasma area under the curve ratio is 2.3 for PFD and as high as 6.5 for its pharmacologically active metabolite, 5-carboxypirfenidone [73]. This indicates that concentrations in the liver are substantially higher than systemic levels, reaching the effective range used in our study. Second, our system, which uses only the parent compound, does not account for the additional antifibrotic activity of these accumulated metabolites. Third, differences in protein binding between plasma (~50–60% bound) and cell culture media (low albumin, high free fraction) mean that the effective free drug concentration in vivo is lower than the total plasma Cmax, narrowing the gap with in vitro nominal concentrations [74]. Finally, the concentrations used here are consistent with the established range in prior foundational in vitro studies on HSCs [33,75]. Notably, our previous study of rat and human HSC metabolism reported that human aHSCs exhibit greater sensitivity to metabolic inhibitors than their rat counterparts, suggesting that our rat-based system may underestimate rather than overestimate the therapeutic potential of metabolic targeting in human aHSCs [76]. Nevertheless, we acknowledge that translating dose-responses to clinical scenarios is complex. Future in vivo studies are essential to validate the clinical relevance of the metabolic mechanisms described herein.

## 5. Conclusions

In summary, our work deciphers a novel metabolic mechanism for PFD, establishing a critical link between its therapeutic action and the suppression of the ATF4 signaling axis. This suppression not only curtails the metabolic pathways necessary for HSC activation but also sensitizes these cells to ferroptotic death. This study provides a preclinical rationale for a new combination therapy that exploits this induced vulnerability. Future research should focus on elucidating the precise molecular interaction by which PFD leads to the inhibition of ATF4 nuclear accumulation. Additionally, exploring whether other antifibrotic compounds converge on this same metabolic axis and screening for more potent and specific combination therapies targeting HSC ferroptosis will be vital for developing next-generation antifibrotic strategies.

## Data Availability

The original contributions presented in this study are included in the article/Supplementary Material. Further inquiries can be directed to the corresponding author.

## Supplementary Materials

The following supporting information can be downloaded at: https://www.mdpi.com/article/10.3390/antiox15050552/s1, Table S1: List of primary antibodies; Table S2: List of primers and probes; Figure S1: Expression patterns of key ferroptosis regulators across HSC subpopulations.

## Abbreviations

The following abbreviations are used in this manuscript:

4-HNE	4-Hydroxynonenal
4-PBA	4-Phenylbutyrate
AIFM1	Apoptosis-inducing factor mitochondria-associated 2
ATF4	Activating transcription factor 4
ATP	Adenosine triphosphate
BDL	Bile duct ligation
CCl4	Carbon tetrachloride
DNP	Dinitrophenol
ECM	Extracellular matrix
ERA	Erastin
Fer-1	Ferrostatin-1
FTH1	Ferritin heavy chain 1
GCH1	GTP cyclohydrolase 1
GLS1/2	Glutaminase 1/2
GPX4	Glutathione peroxidase 4
GSEA	Gene set enrichment analysis
GSH	Glutathione
GSSG	Glutathione disulfide
H2DCFDA	2′,7′-dichlorodihydrofluorescein diacetate
HSC	Hepatic stellate cells
LIP	Labile iron pool
NAC	N-acetylcysteine
OCR	Oxygen consumption rate
P5CS	Pyrroline-5-carboxylate synthase
PFD	Pirfenidone
ROS	Reactive oxidative stress
SHMT2	Serine hydroxymethyltransferase 2
TGF-β	Transforming growth factor-beta
