# Supplementary material for: Pirfenidone Sensitizes Hepatic Stellate Cells to Ferroptosis by Reprogramming Glutamine and Serine Metabolism for GSH Depletion
Source: Antioxidants (Basel). 2026 Apr 26;15(5):552. doi: 10.3390/antiox15050552 (PMC13203578; doi:10.3390/antiox15050552)
Supplement: Supplementary file 1 [file antioxidants-15-00552-s001.zip › antioxidants-4248431-supplementary.pdf]

Table S1. List of primary antibodies

**Antibodies**	**Supplier**	**Identifier**
Collagen type I	SouthernBiotech	1310-01
α-SMA	Sigma-Aldrich	A5228
GLS1	Abcam	Ab93434
SHMT2	Cell Signaling	33443
P5CS	Thermo Fisher Scientific	PA5-52546
4-HNE	R&D Systems	MAB3249
GAPDH	Calbiochem	CB1001

Table S2. List of primers and probes

**Taqman primers and probes**			
**Gene**	**Sense (5'-3')**	**Antisense (5'-3')**	**Probe (5'-3')**
Col1a1 (rat)	TGGTGAACGTGGTGTACAAGGT	CAGTATCACCCTTGGCACCAT	TCCTGCTGGTCCCCGAGGAAACA
Acta2 (rat)	GCCAGTCGCCATCAGGAAC	CACACCAGAGCTGTGCTGTCTT	CTTCACACATAGCTGGAGCAGCTTCTCGA
Mmp9 (rat)	CCCTCTGCATGAAGACGACAT	GGAGGTGCAGTGGGACACA	TCCAGCATCTGTATGGTCGTGGCTCTAAAC
36B4 (rat)	GCTTCATTGTGGGAGCAGACA	CATGGTGTTCTTGCCCATCAG	TCCAAGCAGATGCAGCAGATCCGC

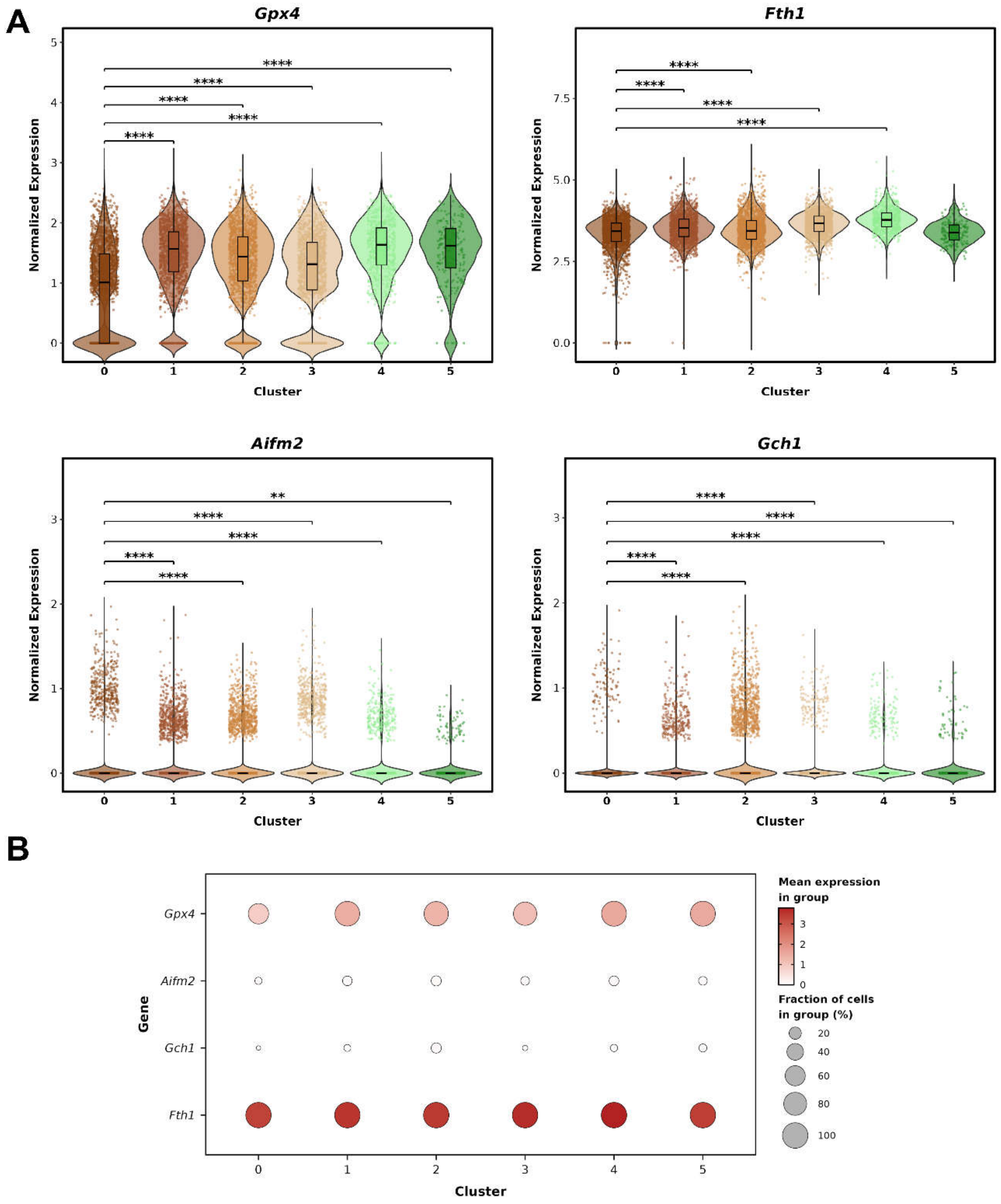

Figure S1. Expression patterns of key ferroptosis regulators across HSC subpopulations. A single-cell RNA-sequencing dataset (GSE171904) was analyzed to examine the expression of ferroptosis-related genes in murine HSC clusters from mice treated with a vehicle control (Oil), carbon tetrachloride (CCl4), or subjected to bile duct ligation (BDL). (**A**) Violin plots showing the distribution of expression levels for key ferroptosis suppressor genes across HSC clusters. Statistical significance of differential expression between cluster 0 and other clusters is indicated above each comparison. (**B**) Dot plot visualizing the expression of ferroptosis regulator genes across HSC clusters. The dot size corresponds to the percentage of cells in the cluster expressing the gene, and the color intensity reflects the average expression level. *$p < 0.05$, **$p < 0.01$, *** $p < 0.001$, **** $p < 0.0001$.
